# Application of a virtual simulation platform combined with the presentation-assimilation-discussion class in human morphology practical teaching: a quasi-experimental study

**DOI:** 10.1186/s12909-026-10141-w

**Published:** 2026-08-13

**Authors:** Xiwen Guo, Wenli Yang, Xiaofang Hu, Si Chen, Zhengdong Jiang, Maosheng Liu, Meibing Lan

**Affiliations:** Department of Human Anatomy and Histoembryology, Zunyi Medical University, Zhuhai Campus, Zhuhai, China

**Keywords:** Human Morphology, Virtual simulation platform, Presentation-assimilation-discussion class, Practical teaching, Undergraduate nursing students, Medical education

## Abstract

**Background:**

Human Morphology integrates anatomy, histology, and embryology and requires students to connect macroscopic and microscopic structures. Traditional practical teaching may be constrained by limited specimen availability, restricted classroom time, and passive learning. This study evaluated a teaching model combining a virtual simulation platform with the presentation-assimilation-discussion (PAD) class in Human Morphology practical teaching.

**Methods:**

A quasi-experimental study was conducted among undergraduate nursing students enrolled in 2023 at the Zhuhai Campus of Zunyi Medical University. The traditional teaching group received conventional instructor-led practical teaching, whereas the combined teaching model group received a teaching approach integrating a virtual simulation platform with the PAD class for one semester. Baseline characteristics, final composite course scores, and score distributions were compared between the two groups. Students in the combined teaching model group completed a questionnaire assessing their self-reported perceptions of learning engagement, teamwork, communication, knowledge application, and acceptance of the virtual simulation platform.

**Results:**

Seventy-eight students were included, with 38 in the traditional teaching group and 40 in the combined teaching model group. No statistically significant differences were observed between the groups in the measured baseline characteristics of age or sex. The mean final composite course score was higher in the combined teaching model group than in the traditional teaching group (76.53 ± 11.07 vs. 72.50 ± 10.40), but the difference was not statistically significant (*P* = 0.102). The combined teaching model group showed a shift toward higher score ranges, although the between-group difference in score distribution was also not statistically significant (*P* = 0.545). Among students in the combined teaching model group, questionnaire results showed generally positive perceptions of the teaching model and of the platform’s usefulness, ease of use, and support for self-directed learning.

**Conclusions:**

The combined virtual simulation–PAD teaching model showed potential value in Human Morphology practical teaching. Although no statistically significant improvement in course performance was observed, students generally reported positive perceptions of classroom participation, self-directed learning, teamwork, communication, and use of the virtual simulation platform. Further studies involving larger samples from multiple universities or campuses, objective practical assessments, and longer follow-up are needed.

**Supplementary Information:**

The online version contains supplementary material available at https://doi.org/10.1186/s12909-026-10141-w.

## Introduction

Human Morphology is an important foundational course in medical education. It integrates anatomy, histology, and embryology and focuses on the structure, development, and functional relationships of the human body [1–3]. Compared with traditional curricula in which anatomy, histology, and embryology are taught separately, Human Morphology adopts an organ system-centered approach that integrates macroscopic and microscopic structures and links morphology with function. This integrated approach may help students develop a more systematic understanding of the relationship between human structure and function. The Human Morphology course offered by our teaching team has been recognized as a provincial first-class course in Guizhou Province. Through continuous teaching practice and curriculum development, the course has gradually established an organ system-centered framework for integrated morphology education.

Practical teaching is an essential component of the Human Morphology curriculum. Traditional practical teaching mainly relies on instructor explanation, demonstration of gross specimens, observation of histological sections, and classroom question-and-answer activities. These approaches help students understand spatial structures, tissue organization, and morphology–function relationships. However, their implementation may be limited by the availability and preservation of specimens, restricted observation angles, laboratory opening hours, and limited opportunities for repeated observation. In addition, inadequate pre-class preparation and insufficient integration of theoretical knowledge with practical observation may lead some students to rely heavily on passive reception and mechanical memorization. Expanding practical teaching resources, increasing student participation, and promoting active learning are therefore important priorities in Human Morphology teaching reform [1].

**Fig. 1:**
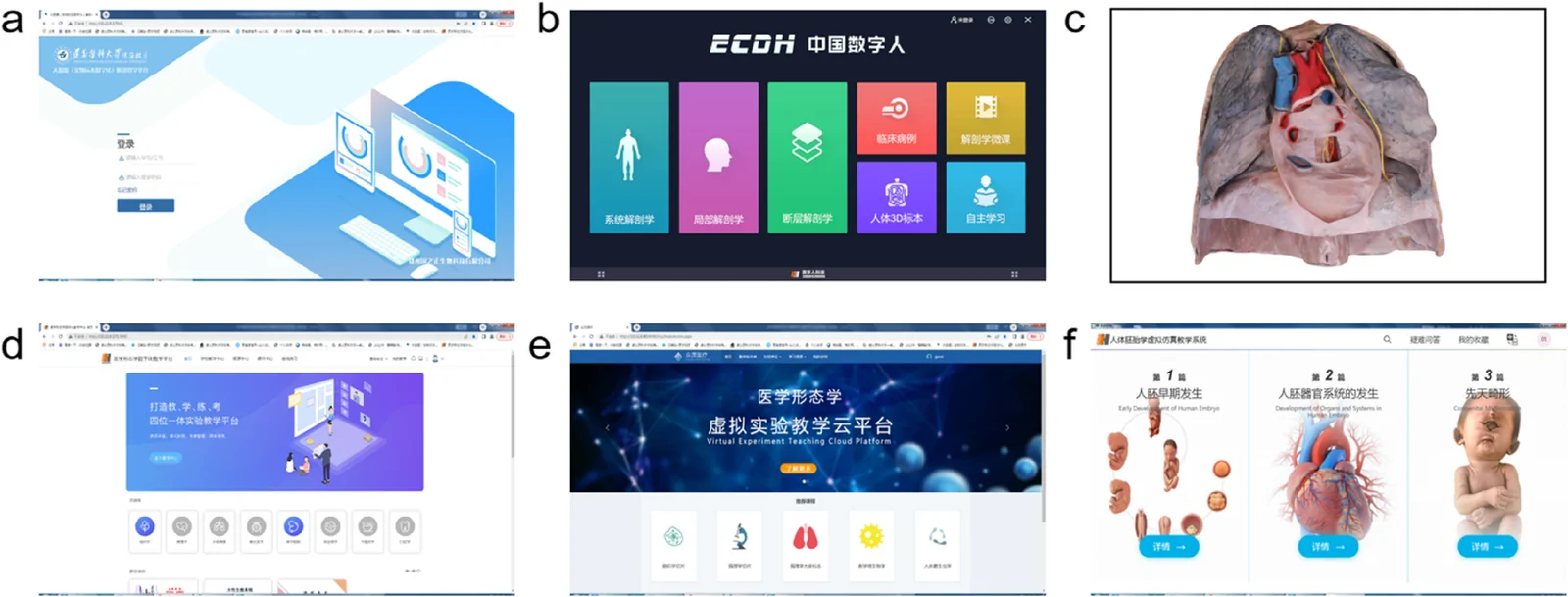
Virtual simulation resources used in Human Morphology practical teaching. The resources included (**a**) a medical morphology digital teaching platform, (**b**) virtual digital human resources, (**c**) a gross specimen image, (**d**) a digital slide platform, (**e**) a virtual simulation teaching platform, and (**f**) embryology-related multimedia resources

The virtual simulation platform used in this course was introduced as a supplementary resource to address some of these limitations. It provides digital slides, virtual digital human resources, gross specimen images, and related multimedia materials that can be accessed repeatedly during pre-class preparation, classroom learning, and after-class review. Figure 1 presents the main platform interfaces and teaching resources used in the course.

In recent years, virtual simulation platforms have been increasingly used in medical education and have provided additional resources for practical teaching [4, 5]. In China, the National Virtual Simulation Experimental Teaching Course Sharing Platform (iLAB-X) has promoted the development and sharing of virtual simulation resources in higher education [6]. These platforms can provide reusable learning materials through digital gross specimens, virtual digital humans, digital slides, and multimedia resources [4–6]. Previous studies have suggested that immersive, collaborative, and interactive virtual environments may support anatomy learning, knowledge retention, student engagement, and acceptance of technology-assisted learning [7–9]. Digital slides and virtual microscopy have also been used in histology teaching to facilitate repeated observation, collaborative learning, active learning, and problem-solving [10–12]. In Human Morphology teaching, such resources may help students connect knowledge across anatomy, histology, and embryology during pre-class preparation, classroom observation, and after-class review.

However, providing digital resources alone may not be sufficient to promote active student participation. The presentation-assimilation-discussion (PAD) class is a teaching model proposed by Professor Xuexin Zhang that organizes learning into three interconnected stages: instructor presentation, individual assimilation, and classroom discussion [13]. The model retains instructor guidance for key concepts while providing students with opportunities for independent preparation, peer communication, knowledge expression, and collaborative learning [13, 14]. Previous studies have applied the PAD class in medical genetics, oral pathology, nursing education, and other courses, with students reporting improvements in learning motivation, self-regulated learning, classroom participation, and learning satisfaction [14–16].

From an educational perspective, the PAD class is consistent with active and constructivist learning principles because students are encouraged to process information independently and construct understanding through discussion, explanation, and problem-solving [17]. Combining a virtual simulation platform with the PAD class may therefore integrate two complementary components: accessible digital learning resources and a structured approach to classroom interaction. This combined model also reflects the principles of blended learning by linking pre-class preparation, face-to-face practical teaching, collaborative discussion, and after-class review [18–20].

**Fig. 2:**
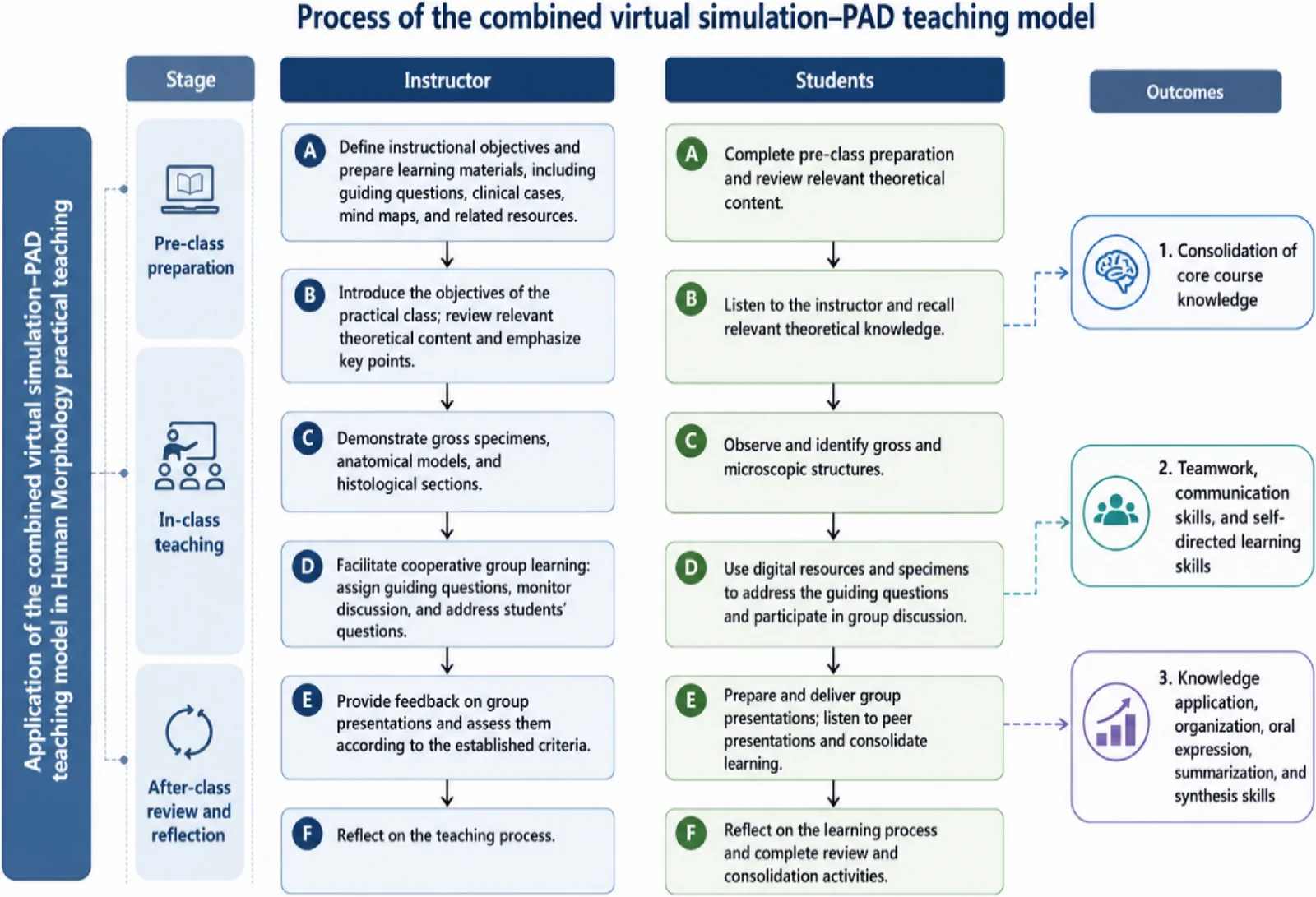
Process of the combined virtual simulation–PAD teaching model in Human Morphology practical teaching. The model integrated pre-class preparation, instructor presentation, specimen demonstration, cooperative group learning, classroom presentation, instructor feedback, and after-class review and reflection

Accordingly, this study aimed to evaluate the application of a combined virtual simulation–PAD teaching model in Human Morphology practical teaching. The study addressed three questions: whether the combined model was associated with differences in students’ final composite course performance compared with traditional teaching; how students perceived and accepted the combined model and the virtual simulation platform; and what implementation challenges should be considered when refining the teaching approach. The overall teaching process is summarized in Fig. 2.

## Methods

### Study design

This quasi-experimental study was conducted during the first semester of the 2023–2024 academic year at the Zhuhai Campus of Zunyi Medical University. The Human Morphology course comprised 106 scheduled teaching hours, including 62 h of theoretical teaching and 44 h of practical teaching.

Two naturally formed classes of first-year undergraduate nursing students were included. One class received the traditional teaching model, whereas the other received the combined virtual simulation–PAD teaching model for one semester. Because the teaching reform was implemented at the class level, individual randomization was not performed.

Both groups followed the same syllabus, teaching objectives, scheduled teaching hours, course content, and assessment framework. They were taught by the same principal instructor and completed the same end-of-semester written theoretical examination. Identical scoring criteria and marking procedures were applied to both groups. The main difference between the groups was the organization of practical teaching.

The study compared students’ final composite course scores and score distributions between the two groups. A questionnaire was also administered to students in the combined teaching model group to assess their self-reported perceptions and acceptance of the teaching model and virtual simulation platform.

### Participants

The participants were first-year undergraduate nursing students enrolled in 2023 at the Zhuhai Campus of Zunyi Medical University. Nursing Class 3 was assigned to the traditional teaching group and included 38 students, whereas Nursing Class 2 was assigned to the combined teaching model group and included 40 students.

The inclusion criteria were as follows: (1) enrollment as an undergraduate nursing student in 2023; (2) completion of all required Human Morphology course activities; (3) completion of the final course assessment; and (4) voluntary participation in the study with written informed consent.

The exclusion criteria were as follows: (1) failure to complete the course or final course assessment; (2) questionnaire responses that were substantially incomplete or internally inconsistent; and (3) unwillingness to participate in the study.

Age and sex were collected as measured baseline characteristics. Because the participants had only recently entered university, university grade point averages, previous university course scores, and other relevant pre-intervention academic performance data were not available for comparison.

### Ethics review and consent to participate

This study was reviewed by the Ethics Committee of Zunyi Medical University, Zhuhai Campus (No. ZHSC-I-[2026]026). The Ethics Committee determined that the study was an educational questionnaire-based study and did not involve human biological research. All students participated voluntarily and provided written informed consent for the use of their anonymized course performance and questionnaire data in this study. Students’ personal information was protected, and all data were anonymized before analysis and used solely for educational research purposes.

### Teaching methods

The two groups followed the same course framework, teaching objectives, scheduled teaching hours, course content, and assessment requirements, as described in subsection 2.1. Both groups were taught by the same principal instructor. The principal difference between the groups was the organization of Human Morphology practical teaching. The traditional teaching group received conventional instructor-led practical teaching, whereas the combined teaching model group received a teaching approach integrating the virtual simulation platform with the PAD class.

#### Traditional teaching group

Before each practical class, students were instructed to review the relevant theoretical content according to the course schedule. During class, the instructor introduced the learning objectives, reviewed key theoretical concepts, and explained the main observational tasks.

The instructor then demonstrated gross specimens, anatomical models, and histological sections and guided students in identifying organ structures, tissue morphology, and their relationships. Students observed the specimens and slides under the instructor’s guidance and recorded the required content.

This teaching approach primarily consisted of instructor explanation, specimen demonstration, guided observation, and classroom question-and-answer activities. It did not include the structured task-divided group discussion and PAD-based presentation procedures used in the combined teaching model group.

#### Combined teaching model group

The combined teaching model group received a teaching approach integrating the virtual simulation platform with the PAD class. The virtual resources included a medical morphology digital teaching platform and a virtual digital human laboratory platform, which provided digital slides, virtual digital human resources, gross specimen images, and multimedia teaching materials for pre-class preparation, in-class observation, and after-class review.

Before each practical class, the instructor distributed key knowledge points, guiding questions, clinical cases, mind maps, and related learning materials through Rain Classroom, a digital teaching platform used to deliver learning resources and support classroom interaction. The 40 students were divided into eight groups of five. Each group leader allocated the 10 guiding questions among the five group members. Students were required to understand their assigned content, prepare clear and comprehensive explanations, and accurately identify the relevant structures.

During class, the instructor first reviewed the relevant theoretical knowledge and demonstrated gross specimens, anatomical models, or histological sections. Students then used physical specimens, histological sections, and virtual simulation resources to discuss the guiding questions and prepare group presentations. Presentation questions were selected by drawing lots during the final 45-minute period. After each presentation, the instructor corrected inaccurate explanations or errors in structural identification, supplemented incomplete content, and re-emphasized key points.

After class, students could continue to use the virtual simulation platform for review and consolidation. The group activities involved task allocation, cooperative learning, and peer explanation but were not designed as a formal Jigsaw cooperative learning intervention.

### Outcome measures

The primary outcome was the final composite course score, ranging from 0 to 100 points. It consisted of an end-of-semester written theoretical examination accounting for 60 points and continuous assessment accounting for 40 points.

Continuous assessment included two specimen-identification assessments (4 points), two electronic assignments (2 points), four low-stakes quizzes (4 points), seven practical reports (7 points), one medical humanities task (1 point), one drawing assignment (2 points), one flipped-classroom activity (6 points), attendance (2 points), and completion of online learning activities through the Xuexitong platform (12 points). The same assessment components, weighting, scoring criteria, and marking procedures were applied to both groups. The mean final composite course score and the distribution of students across different score ranges were compared between the two groups. The four low-stakes quizzes were routine formative assessments and were not designed as equivalent repeated measures for evaluating changes in academic performance over time. The flipped-classroom activity was implemented in both groups using the same assessment criteria and weighting and was not an additional assessment specific to the combined teaching model group.

Questionnaire outcomes were collected only from students in the combined teaching model group and were used to describe their self-reported perceptions of classroom participation, independent thinking, teamwork, communication, knowledge application, and acceptance of the virtual simulation platform.

### Questionnaire design and data collection

The original questionnaire was developed by members of the Human Morphology teaching and research team specifically for course evaluation and teaching feedback. Its design was informed by the characteristics of Human Morphology practical teaching and previous studies evaluating virtual simulation platforms and PAD class teaching [4, 5, 14, 16].

The original questionnaire included single-choice, multiple-choice, and open-ended items concerning course organization, assessment, practical teaching, perceptions of the PAD class, use of virtual learning resources, teamwork, medical humanities education, and suggestions for teaching improvement. In the present study, selected items related to classroom participation, independent thinking, teamwork, communication, knowledge application, and acceptance of the virtual simulation platform were analyzed. The English version of the selected questionnaire items is provided in Additional file 1.

The questionnaire was distributed online through Wenjuanxing, an online questionnaire platform, to students in the combined teaching model group at the end of the course. All 40 students completed the questionnaire, yielding a response rate of 100%, and all submitted questionnaires were included in the descriptive analysis.

The questionnaire was designed as a teaching feedback instrument and was not a previously validated scale. Systematic reliability and validity testing was not performed. Therefore, the questionnaire findings were interpreted as students’ self-reported perceptions and acceptance of the teaching model rather than as objective evidence of competency improvement.

### Statistical analysis

IBM SPSS Statistics version 26.0 was used for statistical analysis. The normality of continuous variables was assessed using the Shapiro–Wilk test. Normally distributed continuous variables, including age and the final composite course score, were expressed as mean ± standard deviation and compared between groups using the independent-samples t-test. Non-normally distributed continuous variables were analyzed using the Mann–Whitney U test.

Categorical variables were expressed as frequencies and percentages and compared using the chi-square test or the Fisher–Freeman–Halton exact test, as appropriate. Questionnaire responses from the combined teaching model group were summarized descriptively as frequencies and percentages, and no between-group statistical comparisons were performed for these data. All statistical tests were two-sided, and a *P* value < 0.05 was considered statistically significant.

## Results

### Baseline characteristics and course performance

A total of 78 first-year undergraduate nursing students were included in the study, with 38 students in the traditional teaching group and 40 students in the combined teaching model group. No statistically significant differences were observed between the two groups in the measured baseline characteristics of age or sex distribution (Table 1).

**Table 1:** Comparison of measured baseline characteristics between the two groups

Item	Traditional teaching group (*n* = 38)	Combined teaching model group (*n* = 40)	*P* value
Age, years	18.53 ± 0.46	18.70 ± 0.51	0.126
Sex			0.567
Male	11 (28.95%)	14 (35.00%)	
Female	27 (71.05%)	26 (65.00%)	

Age is expressed as mean ± standard deviation and was compared using the independent-samples t-test. Sex is expressed as frequency (percentage) and was compared using the chi-square test

The mean final composite course score was higher in the combined teaching model group than in the traditional teaching group (76.53 ± 11.07 vs. 72.50 ± 10.40), but the difference was not statistically significant (*P* = 0.102). Regarding score distribution, the combined teaching model group had higher proportions of students scoring 80–89 and 90–100 and a lower proportion of students scoring below 60 than the traditional teaching group. However, the overall between-group difference in score distribution was not statistically significant (Fisher–Freeman–Halton exact test, *P* = 0.552) (Table 2).

**Table 2:** Comparison of course performance between the two groups

Group	*n*	Final composite course score	< 60	60–69	70–79	80–89	90–100
Traditional teaching group	38	72.50 ± 10.40	6 (15.79%)	12 (31.58%)	10 (26.32%)	7 (18.42%)	3 (7.89%)
Combined teaching model group	40	76.53 ± 11.07	3 (7.50%)	10 (25.00%)	10 (25.00%)	13 (32.50%)	4 (10.00%)

Final composite course scores are expressed as mean ± standard deviation and were compared using the independent-samples t-test (*P* = 0.102). Score distributions are expressed as frequency (percentage) and were compared using the Fisher–Freeman–Halton exact test (*P* = 0.552). The final composite course score consisted of a written theoretical examination accounting for 60 points and continuous assessment accounting for 40 points

### Students’ perceptions of the PAD class teaching model

All 40 students in the combined teaching model group completed the questionnaire, yielding a response rate of 100%. As shown in Tables 3 and 38 students (95.00%) reported that they liked the PAD class teaching model, and 39 students (97.50%) reported willingness to interact actively with classmates or instructors during class. Thirty-six students (90.00%) reported that they could effectively express their ideas, and 39 students (97.50%) considered the guiding questions helpful for mastering the lesson content.

**Table 3:** Students’ self-reported perceptions of the PAD class teaching model

Item	Yes, *n* (%)	No, *n* (%)
Do you like the PAD class teaching model?	38 (95.00%)	2 (5.00%)
Are you willing to actively interact with classmates or teachers in class?	39 (97.50%)	1 (2.50%)
Can you effectively share your ideas in class?	36 (90.00%)	4 (10.00%)
Do you think the guiding questions helped you master the lesson content?	39 (97.50%)	1 (2.50%)
Do you think this teaching model supports independent thinking?	40 (100.00%)	0 (0.00%)
Do you think this teaching model supports teamwork?	37 (92.50%)	3 (7.50%)
Do you think this teaching model supports oral communication?	39 (97.50%)	1 (2.50%)
Can you complete all classroom tasks during class?	33 (82.50%)	7 (17.50%)
Can you apply what you have learned to solve problems?	34 (85.00%)	6 (15.00%)
Do you think this combined teaching model supports Human Morphology practical teaching?	40 (100.00%)	0 (0.00%)

Regarding self-reported competency development, all 40 students (100.00%) perceived that the teaching model supported independent thinking, 37 students (92.50%) perceived support for teamwork, and 39 students (97.50%) perceived support for oral communication. In addition, 33 students (82.50%) reported that they could complete all classroom tasks, and 34 students (85.00%) reported that they could apply the knowledge they had learned to solve problems.

Overall, students reported high acceptance of the PAD class teaching model and generally positive perceptions of its support for classroom participation, peer interaction, and knowledge expression. However, some students reported difficulties in completing classroom tasks and applying knowledge.

#### Students’ acceptance of the virtual simulation platform

As shown in Table 4, students generally reported high acceptance of the virtual simulation platform. All 40 students (100.00%) considered the platform useful for learning Human Morphology and reported that it helped them expand their knowledge. All students also reported that combining the PAD class with the virtual simulation platform increased their interest in learning.

**Table 4:** Students’ self-reported acceptance of the virtual simulation platform

Item	Yes, *n* (%)	No, *n* (%)
The virtual simulation platform is useful for learning this course.	40 (100.00%)	0 (0.00%)
The teaching approach combining the PAD class with the virtual simulation platform increased my learning interest.	40 (100.00%)	0 (0.00%)
The virtual simulation platform helped me learn Human Morphology and expand my knowledge.	40 (100.00%)	0 (0.00%)
The virtual simulation platform is easy to use.	38 (95.00%)	2 (5.00%)
I can access the virtual simulation platform from the dormitory or study room.	33 (82.50%)	7 (17.50%)
I am willing to use the virtual simulation platform as a supplementary tool for specimen identification.	32 (80.00%)	8 (20.00%)
I can successfully access the virtual simulation platform through the provided website.	36 (90.00%)	4 (10.00%)
I can easily find the specimens I want to study on the virtual simulation platform.	32 (80.00%)	8 (20.00%)
The quality of the specimens, videos, and audio resources on the virtual simulation platform is good.	38 (95.00%)	2 (5.00%)
Integrating the virtual simulation platform into classroom teaching increased my confidence in learning the relevant knowledge and skills.	39 (97.50%)	1 (2.50%)

Regarding platform usability, 38 students (95.00%) considered the platform easy to use, and 38 students (95.00%) rated the quality of the specimens, videos, and audio resources positively. Thirty-nine students (97.50%) reported that integrating the platform into classroom teaching increased their confidence in learning the relevant knowledge and skills.

Regarding access and resource retrieval, 33 students (82.50%) reported that they could access the platform from their dormitory or study room, and 36 students (90.00%) reported successful access through the provided website. Thirty-two students (80.00%) preferred using the virtual simulation platform as a supplementary tool for specimen identification, and the same number reported that they could easily locate the specimens they wished to study.

These findings indicate that students generally perceived the virtual simulation platform as a useful and accessible supplementary resource for pre-class preparation, in-class observation, and after-class review. However, a minority of students reported difficulties with platform access and locating specific resources.

### Students’ feedback on classroom interaction and implementation

As shown in Table 3, most students reported positive perceptions of the combined teaching model in relation to independent thinking, teamwork, oral communication, task completion, and knowledge application.

In the open-ended responses, some students suggested providing more opportunities for classroom presentation, allowing additional time for group discussion, and incorporating more clinical cases. These comments indicate that, although the combined teaching model was generally well accepted, further refinement of task difficulty, discussion time, and presentation opportunities may improve its implementation.

## Discussion

### Principal findings

This study evaluated a combined virtual simulation–PAD teaching model in Human Morphology practical teaching. The mean final composite course score was higher in the combined teaching model group than in the traditional teaching group, and the combined teaching model group had higher proportions of students in the upper score ranges and a lower proportion scoring below 60. However, neither the difference in mean course score nor the difference in score distribution reached statistical significance.

These findings do not provide definitive evidence that the combined teaching model improved academic performance. Nevertheless, they suggest that the model is feasible for use in Human Morphology practical teaching and warrants further evaluation in studies with larger samples and more robust designs.

Students in the combined teaching model group generally reported positive perceptions of the PAD class and the virtual simulation platform. They perceived the model as supporting classroom participation, independent thinking, teamwork, oral communication, and self-directed learning. However, because the questionnaire was administered only to the combined teaching model group and was not a validated scale, these findings should be interpreted as descriptive evidence of student acceptance rather than as proof of superiority over traditional teaching.

### Role of the virtual simulation platform in practical teaching

Human Morphology practical teaching requires students to observe and understand gross specimens, anatomical models, and histological sections. However, traditional laboratory learning may be limited by specimen availability, laboratory opening hours, observation angles, and opportunities for repeated review. The virtual simulation platform extended access to learning resources by providing digital slides, virtual digital human resources, gross specimen images, and multimedia materials that students could use before, during, and after class.

In the present study, students generally reported that the platform was useful, easy to use, and supportive of repeated observation and self-directed learning. Previous studies have shown that virtual simulation platforms can support practical teaching and facilitate students’ understanding of complex structures and procedures [4, 5]. Digital slides and virtual microscopy may also provide opportunities for repeated observation and collaborative learning [10, 11]. In addition, immersive virtual anatomy environments have shown potential value in anatomy learning, knowledge retention, student engagement, and technology acceptance [7–9]. These findings are broadly consistent with the positive student perceptions observed in the present study.

Nevertheless, the questionnaire findings reflect students’ subjective acceptance rather than objectively measured improvements in knowledge or practical skills. In addition, some students reported difficulties in accessing the platform or locating specific resources. These findings indicate that platform usability, resource organization, and initial user guidance should be considered when implementing virtual simulation resources.

The virtual simulation platform should therefore be regarded as a supplement to, rather than a replacement for, physical specimens, anatomical models, and histological sections. Its main value may lie in extending opportunities for pre-class preparation, repeated observation, and after-class review while preserving direct practical experience with physical teaching materials.

### Role of the PAD class in promoting active learning and knowledge assimilation

Traditional practical teaching mainly relies on instructor explanation, demonstration, and guided observation. Although this approach supports systematic knowledge delivery, opportunities for structured peer discussion and student explanation may be limited. The PAD class organizes teaching into presentation, assimilation, and discussion, thereby retaining instructor guidance while providing students with opportunities for independent preparation, collaborative learning, and classroom expression.

In the present study, most students reported positive perceptions of the PAD class and considered the guiding questions, group discussion, and classroom presentation helpful for their learning. Students also perceived that the model supported independent thinking, teamwork, and oral communication. These findings are generally consistent with previous studies applying the PAD class in oral pathology, medical genetics, and nursing education [14–16].

The group activities in this study incorporated task allocation, cooperative preparation, peer explanation, and classroom presentation, although they were not designed as a formal Jigsaw cooperative learning intervention. Students may have required time to become familiar with these activities and to develop confidence in explaining knowledge to their peers. This adaptation process may have influenced classroom participation and course performance.

In Human Morphology practical teaching, the PAD class may help students move beyond passive observation by requiring them to interpret structures, organize knowledge, and explain their understanding. However, the present questionnaire findings represent students’ self-reported perceptions, and further studies using objective measures are needed to determine whether the model improves knowledge application or practical competency.

### Complementary roles of the combined teaching model

The virtual simulation platform and the PAD class address different aspects of Human Morphology practical teaching. The virtual simulation platform expands access to reusable visual learning resources, whereas the PAD class provides a structured process for preparation, discussion, explanation, and feedback. Their combination links pre-class preparation, in-class observation and discussion, and after-class review.

From an educational perspective, the combined model is consistent with active learning and constructivist learning principles because students are encouraged to observe, interpret, discuss, and explain knowledge rather than only receive information passively [17]. The integration of digital resources with face-to-face teaching also reflects the principles of blended learning in medical education [18, 19].

Human Morphology practical teaching has strong experiential characteristics, as students need to understand anatomical and histological structures through observation, comparison, discussion, and reflection [20]. In the combined model, virtual resources provided additional opportunities for repeated observation, while the PAD class created opportunities for peer communication and instructor feedback.

However, the higher mean course score observed in the combined teaching model group did not reach statistical significance. Therefore, the present findings do not establish that combining the two approaches improves academic performance. Rather, they suggest that the model is feasible and positively perceived by students and may provide a practical framework for integrating digital resources with structured classroom interaction. Recent studies have also reported the application of blended virtual microscopy in histology laboratory teaching and virtual simulation courses in undergraduate anatomy education, further supporting the continued evaluation of digital resources as supplements to practical teaching [21, 22].

### Implementation challenges and directions for improvement

Although students generally reported positive perceptions of the combined teaching model, several implementation challenges were identified. First, 17.50% of students reported that they could not complete all classroom tasks, and 15.00% reported difficulty applying the knowledge they had learned to solve problems. The number and difficulty of the guiding questions should therefore be adjusted to students’ prior knowledge and the available classroom time.

Second, differences in learning ability and participation may arise among groups. Some students may focus mainly on their assigned questions, while others may participate less actively when they are not selected to present. Future teaching could use instructor-guided grouping, rotating group roles, random questioning, and supplementary explanations to encourage broader participation and ensure that all students engage with the key learning content.

Third, students may require an adaptation period to become familiar with task allocation, cooperative preparation, peer explanation, and classroom presentation. Clearer instructions, examples of effective presentations, and gradual increases in task complexity may help students adapt to the teaching model.

Finally, implementation of the virtual simulation platform requires appropriate technical guidance and resource organization. Brief platform-use training should be provided at the beginning of the course, and expectations for pre-class preparation, in-class use, and after-class review should be clearly communicated. Platform activities may also be linked more closely with formative assessment to encourage effective use rather than simple task completion.

Future refinements should therefore focus on balancing task difficulty and classroom time, improving group organization, supporting students’ adaptation to cooperative learning, and strengthening guidance on the use of virtual resources.

### Limitations

This study has several limitations. First, it was a single-center quasi-experimental study involving only two naturally formed classes, with one class assigned to each teaching group. Individual randomization was not performed, and pre-intervention academic performance data were unavailable. Therefore, possible class-level differences and differences in prior academic ability could not be fully assessed.

Second, although both groups were taught by the same principal instructor and followed the same syllabus, teaching hours, assessment framework, and scoring procedures, the instructor could not be blinded to group allocation. In addition, individual time spent on pre-class preparation, virtual platform use, and after-class review was not recorded, and unmeasured differences in time on task may have influenced the outcomes.

Third, the study lasted for only one semester and did not include equivalent repeated assessments, long-term follow-up, or a separate standardized practical examination. Therefore, changes in performance over time, long-term knowledge retention, and practical competency could not be evaluated.

Finally, the questionnaire was administered only to the combined teaching model group and was not a validated scale. The self-reported findings may have been affected by social desirability bias [23, 24] and should be interpreted as evidence of student perceptions rather than objective competency improvement.

Future studies should include larger samples of students from different health-related academic programs and multiple universities or campuses, together with pre-intervention academic data, learning-time records, standardized practical assessments, and longer follow-up.

## Conclusions

The combined virtual simulation–PAD teaching model was feasible for use in Human Morphology practical teaching and was generally well accepted by students. Although the combined teaching model group had a higher mean final composite course score and a greater proportion of students in the upper score ranges, the between-group differences were not statistically significant.

Students reported positive perceptions of the model in relation to classroom participation, self-directed learning, teamwork, oral communication, and use of the virtual simulation platform. However, these findings were based on self-reported questionnaire data and should not be interpreted as objective evidence of competency improvement.

Further studies involving larger samples of students from different health-related academic programs and multiple universities or campuses, standardized practical assessments, and longer follow-up are needed to evaluate the effects of the combined teaching model on academic performance, knowledge retention, and practical competency.

## Supplementary Information

Supplementary Material 1.

Supplementary Material 2.

## Data Availability

The datasets generated and/or analyzed during the current study are not publicly available because they contain student educational records and questionnaire data, but de-identified data may be available from the corresponding author upon reasonable request and with permission from the relevant institution.

## References

[1] Santos VA, Barreira MP, Saad KR. Technological resources for teaching and learning about human anatomy in the medical course: systematic review of literature. Anat Sci Educ. 2022;15:403–19. 10.1002/ase.2142. 34664384

[2] Cheng X, Chan LK, Li H, Yang X. Histology and embryology education in China: the current situation and changes over the past 20 years. Anat Sci Educ. 2020;13:759–68. 10.1002/ase.1956. 32162490

[3] Dennis JF, Creamer BA. Destination integration: linking physiology, histology, and embryology content in foundational sciences. Front Physiol. 2023;14:1236562. 10.3389/fphys.2023.1236562. PMC10538285 37781227

[4] Lu J, Yang X, Zhao W, Lin J. Effect analysis of a virtual simulation experimental platform in teaching pulpotomy. BMC Med Educ. 2022;22:760. 10.1186/s12909-022-03836-3. PMC9639308 36345029

[5] Sun L, Liu D, Lian J, Yang M. Application of flipped classroom combined with virtual simulation platform in clinical biochemistry practical course. BMC Med Educ. 2023;23:771. 10.1186/s12909-023-04735-x. PMC10577961 37845661

[6] Zhu H, Xu J, Wang P, Liu H, Chen T, Zhao Z, et al. The status of virtual simulation experiments in medical education in China: based on the National Virtual Simulation Experiment Teaching Center (iLAB-X). Med Educ Online. 2023;28:2272387. 10.1080/10872981.2023.2272387. PMC10984652 37883485

[7] Kadri M, Boubakri FE, Kaghat FZ, Azough A, Zidani KA. IVAL: Immersive Virtual Anatomy Laboratory for enhancing medical education based on virtual reality and serious games, design, implementation, and evaluation. Entertain Comput. 2024;49:100624. 10.1016/j.entcom.2023.100624.

[8] Kadri M, Boubakri FE, Hwang GJ, Kaghat FZ, Azough A, Zidani KA. C-IVAL: a longitudinal study of knowledge retention and technology acceptance in collaborative virtual reality-based medical education. IEEE Access. 2025;13:16055–71. 10.1109/ACCESS.2024.3523860.

[9] Kadri M, Boubakri FE, Teo T, Kaghat FZ, Azough A, Zidani KA. Virtual reality in medical education: effectiveness of Immersive Virtual Anatomy Laboratory (IVAL) compared to traditional learning approaches. Displays. 2024;85:102870. 10.1016/j.displa.2024.102870.

[10] Mione S, Valcke M, Cornelissen M. Evaluation of virtual microscopy in medical histology teaching. Anat Sci Educ. 2013;6:307–15. 10.1002/ase.1353. 23463716

[11] Triola MM, Holloway WJ. Enhanced virtual microscopy for collaborative education. BMC Med Educ. 2011;11:4. 10.1186/1472-6920-11-4. PMC3037351 21269474

[12] Tian Y, Xiao W, Li C, Liu Y, Qin M, Wu Y, et al. Virtual microscopy system at Chinese medical university: an assisted teaching platform for promoting active learning and problem-solving skills. BMC Med Educ. 2014;14:74. 10.1186/1472-6920-14-74. PMC4000431 24712715

[13] Zhang X. PAD class: a new attempt in university teaching reform. Fudan Educ Forum. 2014;12:5–10. 10.13397/j.cnki.fef.2014.05.002.

[14] Zhai J, Dai L, Peng C, Dong B, Jia Y, Yang C. Application of the presentation-assimilation-discussion class in oral pathology teaching. J Dent Educ. 2022;86:4–11. 10.1002/jdd.12767. 34403494

[15] Yang YL, Luo L, Qian Y, Yang F. Cultivation of undergraduates’ self-regulated learning ability in medical genetics based on PAD class. Yi Chuan. 2020;42:1133–9. 10.16288/j.yczz.20-186. 33229319

[16] Wen H, Zhang R, Zhou Z, Hong M, Huang Z, Jiang Y, et al. Comparison of lecture-based learning with presentation-assimilation-discussion method in occupational bloodborne exposure education of nursing students, a randomised trial. BMC Nurs. 2024;23:702. 10.1186/s12912-024-02365-2. PMC11441161 39343940

[17] Dong H, Lio J, Sherer R, Jiang I. Some learning theories for medical educators. Med Sci Educ. 2021;31:1157–72. 10.1007/s40670-021-01270-6. PMC8368150 34457959

[18] Vallée A, Blacher J, Cariou A, Sorbets E. Blended learning compared to traditional learning in medical education: systematic review and meta-analysis. J Med Internet Res. 2020;22:e16504. 10.2196/16504. PMC7445617 32773378

[19] Cook DA, Levinson AJ, Garside S, Dupras DM, Erwin PJ, Montori VM. Internet-based learning in the health professions: a meta-analysis. JAMA. 2008;300:1181–96. 10.1001/jama.300.10.1181. 18780847

[20] Yardley S, Teunissen PW, Dornan T. Experiential learning: AMEE Guide 63. Med Teach. 2012;34:e102–15. 10.3109/0142159X.2012.650741. 22289008

[21] Zhang Y, Li C, Zhou C. Improving student learning outcomes and perception through a blended learning strategy based on virtual microscopy for teaching a histology laboratory course. Adv Physiol Educ. 2025;49:79–86. 10.1152/advan.00058.2024. 39570413

[22] Li A, Ma L, Peng R, Shi J, Zhang M, Zheng M, et al. Efficacy of an Imaging Anatomy Virtual Simulation Experiment Course in Undergraduate Medical Education: Nonrandomized Controlled Trial. JMIR Med Educ. 2026;12:e80393. 10.2196/80393. PMC13171472 42127315

[23] van de Mortel TF. Faking it: social desirability response bias in self-report research. Aust J Adv Nurs. 2008;25:40–8.

[24] Latkin CA, Edwards C, Davey-Rothwell MA, Tobin KE. The relationship between social desirability bias and self-reports of health, substance use, and social network factors. Addict Behav. 2017;73:133–6. 10.1016/j.addbeh.2017.05.005. PMC5519338 28511097

