# Supplementary material for: Application of a virtual simulation platform combined with the presentation-assimilation-discussion class in human morphology practical teaching: a quasi-experimental study
Source: BMC Med Educ. 2026 Aug 13;26:1826. doi: 10.1186/s12909-026-10141-w (PMC13749875; doi:10.1186/s12909-026-10141-w)
Supplement: Supplementary file 1 [file 12909_2026_10141_MOESM1_ESM.pdf]

Review opinions of the ethics committee of the applicant's unit

Research project name	Application of Virtual Simulation Experimental Platform in Human Morphology Experimental Teaching
Classification of research projects	☐ Scientific research topic (☐ Basic research ☐ Applied basic research ☐ Clinical research☐ Pharmacy ☐ Other)<br>☑ other
Research materials and experimental types	☑ Collection of human specimens (☐ Blood ☐ Urine ☐ Feces ☐ Sputum ☑ Others) ☐ Collection of animal specimens (☐ Blood ☐ Cells ☐ Tissue ☐ Other) ☐ Animal experiments ☐ Human experiments
Review documents	1. Medical Ethics Review Form ☐2. Informed consent form ☐<br>3. Animal Ethics Review Form ☐4. Project Application Form ☐<br>Other documents ☐
Review documents	☐ Meeting review☑ Letter review☐ Quick review

Review conclusion :

This subject matter is investigated by questionnaire survey and doesn't involve human biology.

Signature of Ethics Committee Member:	Signature of Chairman/Vice Chairman:<br><br>Ethics Committee of the applicant's unit (seal)<br><br>Date: May 8, 2026

NO. ZHSC-1-[2026]026
