# Supplementary material for: Application of a virtual simulation platform combined with the presentation-assimilation-discussion class in human morphology practical teaching: a quasi-experimental study
Source: BMC Med Educ. 2026 Aug 13;26:1826. doi: 10.1186/s12909-026-10141-w (PMC13749875; doi:10.1186/s12909-026-10141-w)
Supplement: Supplementary file 2 [file 12909_2026_10141_MOESM2_ESM.pdf]

Title:

Questionnaire on students' perceptions of the PAD class teaching model and the virtual simulation experimental platform in human morphology experimental teaching

Instructions:

This questionnaire was developed by the research team for teaching feedback and research analysis. It was distributed online through Wenjuanxing at the end of the course. The selected items listed below were analyzed in the present study. Students answered each item according to their actual learning experience.

Part 1. Students' perceptions of the PAD class teaching model

1. Do you like the PAD class teaching model?

A. Yes

B. No

2. Are you willing to actively interact with classmates or teachers in class?

A. Yes

B. No

3. Can you effectively share your ideas in class?

A. Yes

B. No

4. Do you think the guiding questions helped you master the lesson content?

A. Yes

B. No

5. Do you agree that this teaching method can improve independent thinking?

A. Yes

B. No

6. Do you agree that this teaching method can improve teamwork?

A. Yes

B. No

7. Do you agree that this teaching method can improve oral expression?

A. Yes

B. No

8. Can you complete all classroom tasks during class?

A. Yes

B. No

9. Can you apply what you have learned to solve problems?
A. Yes
B. No

10. Do you think this experimental teaching model improves human morphology teaching?
A. Yes
B. No

Part 2. Students' acceptance of the virtual simulation experimental platform

11. The virtual simulation experimental platform is useful for learning this course.
A. Yes
B. No

12. The teaching approach combining the PAD class and the virtual simulation experimental platform increased my learning interest.
A. Yes
B. No

13. The virtual simulation experimental platform helped me learn human morphology and expand my knowledge.
A. Yes
B. No

14. The virtual simulation experimental platform is easy to use.
A. Yes
B. No

15. I can log in to the virtual simulation experimental platform from the dormitory or study room.
A. Yes
B. No

16. Compared with traditional laboratories, I am more willing to use the virtual simulation experimental platform to support specimen recognition.
A. Yes
B. No

17. I can successfully log in to the virtual simulation experimental platform through the provided website.
A. Yes
B. No

18. I can easily find the specimens I want to study on the virtual simulation experimental platform.
A. Yes
B. No

19. The quality of specimens, videos, and audio on the virtual simulation experimental platform is good.
A. Yes
B. No

20. Integrating the virtual simulation experimental platform into classroom teaching increased my confidence in mastering knowledge and skills.
A. Yes
B. No

Note:
The original course evaluation questionnaire included broader items on course and examination feedback, experimental teaching design, learning resource use, medical humanities teaching, teamwork, and open-ended suggestions. The present study analyzed selected items related to students' perceptions of the PAD class teaching model and the virtual simulation experimental platform.
